# Study on the physicochemical indexes, nutritional quality, and flavor compounds of *Trichiurus lepturus* from three representative origins for geographical traceability

**DOI:** 10.3389/fnut.2022.1034868

**Published:** 2022-11-01

**Authors:** Shitong Wang, Pingya Wang, Yiwei Cui, Weibo Lu, Xuewei Shen, Huimin Zheng, Jing Xue, Kang Chen, Qiaoling Zhao, Qing Shen

**Affiliations:** ^1^Collaborative Innovation Center of Seafood Deep Processing, Zhejiang Province Joint Key Laboratory of Aquatic Products Processing, Institute of Seafood, Zhejiang Gongshang University, Hangzhou, China; ^2^Zhoushan Institute of Food & Drug Control, Zhoushan Institute of Calibration and Testing for Quality and Technology Supervision, Zhoushan, China

**Keywords:** *Trichiurus lepturus*, geographical traceability, nutritional components, fatty acid, volatile flavor substances

## Abstract

Trichiurus lepturus (hairtail) is an important economic component of China’s marine fishing industry. However, due to the difficulty in identifying the appearance of hairtail from different geographical distributions, hairtails with geographical indication trademarks were imitated by general varieties. In this study, the texture characteristics, color, basic nutrients, amino acids, mineral, fatty acids, and volatile flavor substances were used as indicators for multivariate statistical analysis to determine whether three origins of hairtails from the habitats of Zhoushan (East China Sea, T.Z), Hainan (South China Sea, T.N), and Qingdao (Yellow Sea, T.Q) in the market could be distinguished. The findings revealed that there were significant differences in amino acids composition, mineral composition, fatty acid composition in lipids, and volatile flavor substances among the hairtails of three origins (*P* < 0.05), but no differences in color, texture, protein content. T.Z had moisture, crude fat, essential amino acids (EAA), flavor amino acids (FAA), unsaturated fatty acids (UFA), and docosahexaenoic acids and dicosapentaenoic acids (ΣEPA + DHA) contents of 74.33, 5.4%, 58.25 mg⋅g^–1^, 46.20 mg⋅g^–1^, 66.84 and 19.38%, respectively, and the contents of volatile alcohols, aldehydes and ketones were 7.44, 5.30, and 5.38%, respectively. T.N contains moisture, crude fat, EAA, FAA, UFA and ΣEPA + DHA as 77.69, 2.38%, 64.76 mg⋅g^–1^, 52.44 mg⋅g^–1^, 65.52 and 29.45%, respectively, and the contents of volatile alcohols, aldehydes and ketones as 3.21, 8.92, and 10.98%, respectively. T.Q had the contents of moisture, crude fat, EAA, FAA, UFA, and ΣEPA + DHA 79.69, 1.43%, 60.9 mg⋅g^–1^, and 49.42 mg⋅g^–1^, respectively. The contents of unsaturated fatty acid and ΣEPA + DHA were 63.75 and 26.12%, respectively, while the volatile alcohols, aldehydes, and ketones were 5.14, 5.99, and 7.85%, respectively. Partial least squares-discriminant analysis (PLS-DA) multivariate statistical analysis showed that volatile flavor compounds could be used as the most ideal indicators for tracing the source of hairtail. In conclusion, the findings of this study can distinguish the three hairtail origins using some basic indicators, providing ideas for hairtail geographical identification.

## Introduction

Under the pressure of high intensity fishing and the global decline of marine fishery resources, Chinese *Trichiurus lepturus* (hairtail) has maintained high yield, ranking first in the output of single species of marine fishing in China. According to a World Food and Agriculture Organization (FAO) statistical survey, the average annual output of the world’s hairtail from 2005 to 2016 was as high as 1.31 million tonnes, with approximately 80 percent caught in China’s oceans.

Chinese hairtail is distributed in the Yellow Sea, East China Sea, Bohai Sea and South China Sea, among which Zhoushan hairtail (*Trichiurus japanicus*, T.Z) in the East China Sea, Qingdao hairtail (*Trichiurus japanicus*, T.Q) in the Yellow Sea and Nanhai hairtail (*Trichiurus nanhaiensis*, T.N) in the South China Sea are the most prevalent in the market in China. China’s sea area is vast and the water quality conditions such as temperature, salinity, water mass, dissolved oxygen, substrate type, and feed organisms, varies from sea to sea, so the quality of hairtail caught in different sea areas will also vary (1). The population of T.Z is distributed along the mixed waters of the coastal water system, the Taiwan warm current, and the Yellow Sea cold water mass, and the continuous injection of continental current brings a large amount of plankton and seawater nutrients to the habitat. Taking these factors into account, T.Z have been considered preferable in fish quality and taste compared to those from other regions, and it has become the owner of China’s first seafood geographical indication certification trademark. However, due to the morphology of hairtail is complex and changeable, and the differences in fishing season, location, growth stage and sample specifications, the morphological identification may get wrong results. In addition, the commercial hairtail is mostly sold in the form of a segment, which further aggravates the difficulty of traceability of hairtail. This phenomenon can lead to seafood fraud, and the consequences of intentional species substitution and mislabeling may lead to some illegal profiting behaviors (2), which damages the interests of consumers. Therefore, necessitating research into product-specific and generic analytical fish product traceability systems (3).

There have been some detecting methods established for authenticating seafood traceability and identificating seafood mislabeling and fraud. Emerging techniques for seafood authentication include polymerase chain reaction-restriction fragment length polymorphism (PCR-RFLP), real-time PCR, droplet digital PCR, isothermal amplification, PCR-enzyme-linked immunosorbent assay (ELISA), and high-throughput or next-generation sequencing (4, 5). However, the classification accuracy would be unpredictable for genotypic methods due to the DNA contamination or analytes complexity. Recently, lipidomics method has been proven to be an effective tool for seafood provenance due to the unique lipid profile of each source organism (6). Yu et al. reported that lipidomics fingerprints, detected by hydrophilic interaction chromatography/mass spectrometry (HILIC/MS), of rainbow trout and two salmons were distinct and specific (7). Lu et al. established an *in situ* and real-time authentication method that used iKnife rapid evaporative ionization mass spectrometry (REIMS) based lipidomics for distinguishing the seven shrimp species (8). Electronic-nose (e-nose) sensor technology have been primarily used for the purposes of detection, discrimination and recognition of simple and complex gaseous mixture, which has great potential in the application of seafood traceability. Karunathilaka et al. evaluated an e-nose sensor in combination with support vector machine (SVM) modeling for predicting the decomposition state of four types of shllets (9). Wu et al. conducted qualitative and quantitative analyses of flavor substances in fresh hairtail, traditional, and fermented dried hairtail using an electronic nose and headspace solid-phase microextraction (HS-SPME-GC-MS) (10). However, detection instruments for these technology are expensive and not easy to get access, moreover, the data analysis is time-consuming. The solution to the traceability problem of hairtail caught from different sources in the Chinese market has not been reported. There are currently no relevant reports that distinguish different regions hairtail based on basic biological and chemical parameters.

In this study, three different geographic groups of hairtail, including T.Q, T.Z, and T.N were collected and their textural characteristics, color, nutritional components, amino and volatile flavor substances were compared to obtain a comprehensive and systematic understanding of the basic characteristics and nutritional values of hairtail from different origins. Besides, the classification ability of these indicators for the three origin hairtail was compared by multivariate statistical analysis, providing the optimal ideas for tracing to the source of fishing.

## Materials and methods

### Materials and reagents

**FIGURE 1:**
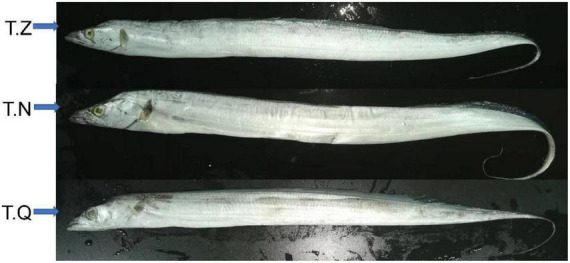
External morphology of three origin hairtail.

As shown in Figure 1, in order to ensure the authenticity of the hairtail, all the hairtail purchased in this experiment were complete hairtail transported by the cold chain. Zhoushan hairtail was purchased from the aquatic products business department of Zhoushan Putuo Jimei (Zhoushan, Zhejiang). Qingdao Hengjie Agricultural Products Co., Ltd. supplied Qingdao hairtail (Qingdao, Shandong). Hainan hairtail was bought from Hainan Wanyue Trading Co., Ltd. (Haikou, Hainan). Merck provided chromatography-grade acetonitrile, chloroform, and methanol (Darmstadt, Germany). Anpel Laboratory Technologies Inc. provided the standard mixture of 37 fatty acid methyl esters (FAMEs) (Shanghai, China). Sinopharm Chemical Reagent Co., Ltd. provided the other analytical grade chemicals and solvents (Shanghai, China).

### Determination of color difference value

A Hunter Lab colorimeter was used to measure the fish color difference values (Lab value) of hairtail lateral lines in the front, middle, and back positions (Colorquest II, Hunter Associates Laboratory Inc., Reston, VA, USA). The hairtail were dissected and skinned, and the muscles (3 × 3 × 2 cm) above the fish’s anterior, middle, and posterior lateral lines were cut to measure the color difference (Lab value), with the average value of the three parts used as the result.

### Texture profile analysis

Texture profile analysis (TPA) was performed with a texture analyzer (TA-XT2i, Stable Micro System, UK) and a TMS P5 flat bottom cylindrical probe, and the samples’ norm was 2 × 2 × 2 cm above the lateral line. Pre-test rate was 1 mm⋅s^–1^; test rate was 1 mm⋅s^–1^; post-test rate was 1 mm⋅s^–1^; deformation rate was 60%; time internal was 5 s; initial test force was 0.01 N; data collection rate was 200 pps. Parallel experiments were carried out with six fish tails per group.

### Determination of water, ash, mineral, crude protein, crude fat, and amino acid contents

Muscle water content was determined using the direct drying method, as specified in GB5009.3–2016. The ash content was determined in accordance with GB5009.4–2016, using the muffle furnace burning method. The crude protein content was determined using an automatic Kjeldahl nitrogen analyzer in accordance with GB5009.5–2016. Soxhlet extraction method for determining crude fat content in accordance with GB5009.6–2016. Mineral content was determined using inductively coupled plasma optical emission spectroscopy (ICP-OES) in accordance with GB5009.268–2016, and the dry digestion method was used for pretreatment. The hydrolysis amino acid method was used to determine the amino acid content in accordance with GB 5009.124–2016.

### Analysis of fatty acids

A modified Folch method (11) was used to extract lipids. In a nutshell, 10 g of minced fish was accurately weighed and distributed in a mixture of chloroform and methanol (30 mL, 2:1, v/v) and distilled water (10 mL), which was then immersed in sonication (53 Hz, 350 W) for 20 min at room temperature. The mixture was then separated into two phases by centrifugation at 8000 rpm for 10 min (Thermo Scientific, Waltham, MA, USA). To remove organic reagents and obtain crude lipids, the lower organic phase was rotary evaporated at 70°C.

Fatty acid methyl esters (FAMEs) were created by esterifying crude lipids (12). In particular, 0.1 g of fish oil was precisely weighed and mixed with a 5 mL potassium hydroxide-methanol solution in a stoppered test tube at 65°C for 20 min. After naturally cooling to room temperature, boron trifluoride-methanol (700 μL, 55–60%) was added to shake at 65°C water bath for 3 min before being removed and naturally cooled to room temperature after 10 min of ultrasonication. The mixture was then extracted and washed with hexane (2 mL) and saturated sodium chloride solution (2 mL). After standing the layers, the supernatant was aspirated, and a small amount of anhydrous sodium sulfate was added to remove the water. For gas chromatography, the supernatant was filtered through a 0.22 μm organic filter (GC).

The FAMEs were analyzed using an Agilent model 7890A capillary GC instrument with an HP-88 capillary column (Agilent Technologies Co. Ltd., Santa Clara, CA, USA) (100% cyanopropyl polysiloxane; 30 m × 0.25 mm, 0.2 μm membrane, Agilent Technologies Co. Ltd). Each time, high-purity nitrogen was used as the carrier gas, with a flow rate of 0.65 mg⋅min^–1^ and a sample volume of 1 μL. The temperature of the flame ionization detector (FID) was set to 250°C, and a gradient heating procedure was used. The temperature was set to 25°C for 5 min, then ramped to 120°C at a rate of 15°C per minute for 1 min, then to 175°C at a rate of 5°C per minute for 5 min, and finally to 220°C in 5 min and held for 5 min (7).

### Determination of flavor substances

The volatile compounds in the three samples were analyzed using a headspace solid-phase microextraction gas chromatography-mass spectrometry (HS-SPME-GC/MS) with a TR-35MS elastic capillary column (30 m × 0.25 mm I.D., 0.25 μm film, Agilent Technologies Co. Ltd., DE, USA). The 4 g sample was placed in a 15 mL headspace vial and balanced in a water bath at 60°C for 20 min. The volatile was then adsorbed for 30 min with a divinylbenzene/carboxyl/polydimethylsiloxane (DVB/CAR/PDMS) extraction head and desorbed in a syringe (250°C, 1 min) at a flow rate of 1 mg⋅min^–1^ of helium (99.99%). The gradient procedure was set as follows: the initial temperature was 40°C, which was maintained for 3 min, then increased to 90°C at 5°C⋅min^–1^ which was maintained for 7 min, then continuously increased to 230°C at 20°C⋅min^–1^ which was maintained for 7 min. Electron bombardment ionization mode 70 eV, source temperature 250°C, interface temperature 280°C, and mass scanning range 33–450 m/z were the mass spectrometry conditions. Quantitative analysis with peak area normalization was used to calculate the relative content of each chemical component.

### Statistical analysis

The experimental data were imported into Microsoft Excel 2016 (Microsoft Corp., Redmond, WA, USA), mean and standard deviation analysis were performed using SPSS version 26 (SPSS Inc., Chicago, IL, USA) that corresponding figures were drawn using OriginPro 2021 (OriginLab Corp., Northampton, MA, USA), and multivariate statistical analysis were processed at https://dev.metaboanalyst.ca/MetaboAnalyst/. When the difference was analyzed using one-way ANOVA, normality, equal variance, and *post hoc* Duncan’s multiple range tests, *P* < 0.05 was considered statistically significant.

## Results and discussion

### Color

The color of fish is the result of long-term natural selection and adaptation to the ecological environment, and it is an essential criterion for assessing the freshness, quality, and acceptability of fish (13). Color can be used to identify hairtail in different sea areas and reflect the variation of their habitat environment to some extent. Therefore, the color of hairtail was studied, and the findings are shown in Table 1. The values of lightness (L*), redness (a*), and yellowness (b*) were obtained by employing a colorimeter with the aim of defining the surface color of the muscle sample. T.Q had the highest L* value (52.33) among these three hairtail samples, which was significantly higher (*P* < 0.05) than that of T.N. This could be explained by the high water content (79.64%) of muscle above the lateral-line of T.Q. Although T.Z has the lowest moisture content (74.81%) of the three hairtail samples, its high levels of oil content (5.38%) increased the intensity of light reflection from the muscle surface, which can explain why there is no statistically significant (*P* > 0.05) difference in the brightness of T.Z moisture. The a* values of hairtails were on a negative scale ranging from –1.71 (T.Z, control) to –1.52 (T.N, control), and the effect of sample origin was not statistically significant (*P* > 0.05). When compared to T.Z (4.44), the b* values of T.N (3.98) and T.Q (3.87) decreased significantly (*P* < 0.05). This variation could be attributed to the higher fat content of T.Z-fed nutrients, which promotes the absorption of pigments like astaxanthin and lutei (14, 15). Finally, the above findings demonstrated that the color of fish is not only determined primarily by genetic genes but is also closely related to its living environment and nutritional status.

**TABLE 1:** The color parameters of hairtails of the three origins.

Samples	L*	a*	b*
T.Z	51.52 ± 1.27^ab^	–1.71 ± 0.13^a^	4.44 ± 0.20^a^
T.N	49.72 ± 0.99^b^	–1.52 ± 0.11^a^	3.98 ± 0.16^b^
T.Q	52.33 ± 1.15^a^	–1.68 ± 0.11^a^	3.87 ± 0.13^b^

^a–c^Mean values in the same column (corresponding to the same parameter) not followed by a common letter differ significantly (P < 0.05). T.Z, Zhoushan hairtail; T.N, Hainan hairtail; T.Q, Qingdao hairtail.

### TPA

TPA is a compression method that is widely used in the determination of food texture, which is an important index for assessing fish freshness and sensory quality. Its theory is to simulate the occlude action of the human mouth cavity by squeezing the sample twice with the texture analyzer probe, hence the name “Two-bite test” (16). The advantage was that it could record the correlation between time and force using mechanical experiments on hairtail samples and obtain multiple physical properties at once, such as hardness, springiness, gumminess, and chewiness, as shown in Table 2. T.Q lump had the highest hardness (5.83 N) compared to T.Z (5.16 N) and T.N (4.34 N), but sea area difference had no significant (*P* > 0.05) effect on the hardness of these hairtails. This result was consistent with the findings of Ayala (17) that hairtail movement ability was similar in the three living environments, resulting in similar muscle fiber diameter and arrangement, and then similar hardness in macroscopically. When it comes to cohesiveness, there were differences in the binding ability between muscle cells due to the three origin hairtail’ homologous motor ability, which revealed that the cohesiveness of T.Z sample (0.44) was slightly better than that of T.Q sample (0.38), but this advantage is not obvious. In the term of springiness, sample T.Q (5.55 mm) was weaker (*P* < 0.05) than samples T.Z (6.72 mm) and T.N (7.64 mm). Springiness is the deformation of a sample caused by an external force that is restored after the force is removed. Among the three main components of fish (myofibrillar protein, sarcoplasmin, and myostromal protein), the myofibrillar protein contains a lot of actin and myosin, and the myostromal protein has a lot of elastin which affects the springiness of hairtail. Gumminess is the property of being cohesive and sticky, whereas chewiness is the sensory “bite” that is positively related to taste. Protein, moisture, and fat content all have an impact on the texture of fish. The higher the fat content of fish, the more chewable it is.

**TABLE 2:** Texture profile analysis of three type hairtail.

Samples	T.Z	T.N	T.Q
Hardness (N)	5.16 ± 1.14^a^	4.34 ± 0.70^a^	5.83 ± 1.86^a^
Cohesiveness (Ratio)	0.44 ± 0.03^a^	0.32 ± 0.03^b^	0.38 ± 0.07^ab^
Springiness (mm)	6.72 ± 0.97^a^	7.64 ± 0.81^a^	5.55 ± 0.37^b^
Gumminess (N)	2.24 ± 0.53^ab^	1.36 ± 0.25^b^	2.53 ± 1.11^a^
Chewiness (mJ)	13.77 ± 3.32^a^	9.87 ± 1.87^a^	12.32 ± 3.91^a^

^a–c^Mean values in the same row (corresponding to the same parameter) not followed by a common letter differ significantly (P < 0.05). T.Z, Zhoushan hairtail; T.N, Hainan hairtail; T.Q, Qingdao hairtail.

### Nutrition

**FIGURE 2:**
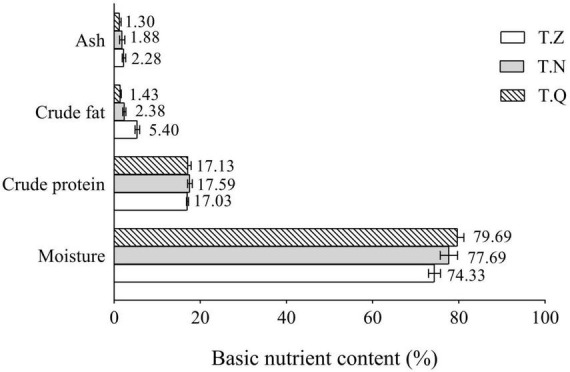
Hairtail muscle nutrient content from three different sources.

Figure 2 depicts the basic nutrient composition values recorded for various hairtail samples. It is worth noting that the moisture and crude lipid content of the hairtail samples differed significantly. T.Q had the highest moisture percentage (79.69%). T.Z had the highest ash content (2.28%). The three samples had approximative protein percentages (17.03–17.59%). The protein content is thought to be determined by the species’ genetic characteristics and unaffected by diet (18). The crude lipid content of hairtail was highest in T.Z (5.40%), followed by T.Q (2.38%), while lowest in T.Q. (1.43%). Normally, increased fat in the fish causes a decrease in moisture content (19), so it seems reasonable to assume that the real differences between the three hairtail were primarily due to fat content variability. T.Z were fatter, whereas the other hairtail were leaner, with the former being preferred by the general consumer due to the great taste and high nutritional qualities found in fat fish.

### Amino acid

Amino acid content and composition are critical indicators for determining the nutritional value of proteins in foods (20). Table 3 shows the composition of 17 amino acids detected after acid hydrolysis of fish from three sources, with the exception of tryptophan, which was not detected because it is completely destroyed during protein acid hydrolysis. There were four flavor amino acids detected (aspartic acid, glutamate, glycine, alanine), seven essential amino acids detected (threonine, valine, methionine, phenylalanine, isoleucine, leucine, lysine), three half-essential amino acids detected (histidine, arginine, cystine), and three non-essential amino acids detected (serine, tyrosine, proline). T.N had a higher total amino acid content of 160.65 mg⋅g^–1^ than T.Q (141.41 mg⋅g^–1^) and T.Z (150.02 mg⋅g^–1^).

**TABLE 3:** The amino acids composition in muscle of hairtail (mg⋅g^–1^).

Amino acid	T.Z	T.N	T.Q
Threonine+	6.06	6.82	6.30
Valine+	5.93	6.60	6.12
Methionine+	6.26	7.00	6.58
Isoleucine+	6.96	7.54	7.30
Leucine+	14.48	15.82	15.04
Phenylalanine+	6.16	7.08	6.60
Lysine+	12.40	13.90	12.96
Histidine*	3.12	3.64	3.30
Arginine*	17.56	20.92	18.98
Aspartic Acid^&	12.84	14.54	13.58
Glutamate^&	20.28	22.12	21.94
Glycine^&	5.32	7.00	5.70
Alanine^&	7.76	8.78	8.20
Proline&	4.10	4.82	4.54
Serine&	5.84	6.82	6.20
Cystine	0.10	0.14	0.10
Tyrosine	6.24	7.02	6.58
ΣTAA	141.41	160.56	150.02
ΣEAA	58.25	64.76	60.90
ΣHEAA	20.68	24.56	22.28
ΣFAA	46.20	52.44	49.42
ΣNEAA	56.14	64.08	60.16
EAA/TAA	0.41	0.40	0.41
EAA/(HEAA + NEAA)	0.76	0.73	0.74
FAA/TAA	0.33	0.33	0.33

+ Means essential amino acids (EAA); * means half-essential amino acids (HEAA); ^ means flavor amino acids (FAA); & means non-essential amino acids (NEAA). TAA, total amino acids; T.Z, Zhoushan hairtail; T.N, Hainan hairtail; T.Q, Qingdao hairtail.

T.N had the highest essential amino acid content (64.76 mg⋅g^–1^), half-essential amino acid content (24.56 mg⋅g^–1^), umami amino acid content (52.44 mg⋅g^–1^), and non-essential amino acid content (64.08 mg⋅g^–1^) when compared to the other two hairtail samples. Despite being geographically diverse, the ratio of essential amino acids to total amino acids in these hairtail samples was close to 0.41, which was consistent with the discovery of Xu et al. (21). in Zhoushan hairtail muscle and fully conformed to FAO/WHO standard requirements (nearly 0.40). The essential amino acid to non-essential amino acid ratio ranged from T.N (0.73) to T.Q (0.76), which was higher than the FAO/WHO reference protein value of greater than 0.60. In flavor amino acids, glutamic acid and aspartic acid were the most umami, with glutamic acid being the most umami in particular, whereas glycine and alanine were the sweetest. T.N sample had a higher content of flavorful amino acids (52.44 mg⋅g^–1^), with 20.12 mg⋅g^–1^ glutamate. However, the ratio of flavorful amino acids to total amino acids of the three hairtail samples was 0.33, indicating that these hairtail had a consistent sense of delicious taste.

Table 4 shows the amino acid score (AAS), chemical score (CS), and essential amino acid score (EAAI) calculated and evaluated using the FAO/WHO amino acid scoring standard model and the egg protein scoring standard model. According to the AAS model, the first limiting amino acid in hairtail muscle samples was valine, and the second limiting amino acid was threonine; similarly, the first and second limiting amino acids in hairtail muscle samples were valine and methionine + cystine, respectively. In other words, the valine, threonine, methionine, and cystine in hairtail muscle protein were deficient when compared to other essential amino acids. T.Z [72.68 mg⋅(g⋅pro)^–1^], T.N [78.93 mg⋅(g⋅pro)^–1^], and TQ [75.52 mg⋅(g⋅pro)^–1^] had slightly higher lysine content than egg protein mode [70 mg⋅(g⋅pro)^–1^], but significantly higher than FAO/WHO mode [55 mg⋅(g⋅pro)^–1^]. Because lysine is the first limiting amino acid in wheat, corn, and other cereals, eating hairtail can compensate for the lack of lysine, greatly improving protein digestion and absorption (16). T.N (82.72) had a higher EAAI value than T.Z (76.95) and T.Q (79.84), indicating that the overall essential amino acids in T.N protein were more in line with human nutritional requirements, which may be influenced by physiological state, culture environment, water area, and water salinity. Except for the first and second limiting amino acids, the AAS value of essential amino acids was all greater than one, demonstrating that the essential amino acid composition of these three origin hairtail was in a suitable proportion and the nutritional value was abundant.

**TABLE 4:** Essential amino acid content, AAS, CS, and EAAI in hairtail muscle (mg⋅(g⋅pro)^–1^).

EAA	FAO/WHO pattern	Egg protein pattern	T.Z	T.N	T.Q	AAS			CS		
						T.Z	T.N	T.Q	T.Z	T.N	T.Q
Isoleucine	40.00	54.00	40.80	42.82	42.54	1.02	1.07	1.06	0.76	0.79	0.79
Leucine	70.00	86.00	84.88	89.84	87.65	1.21	1.28	1.25	0.99	1.04	1.02
Lysine	55.00	70.00	72.68	78.93	75.52	1.32	1.44	1.37	1.04	1.13	1.08
Methionine + Cystine	35.00	57.00	37.28	40.55	38.93	1.07	1.16	1.11	0.65	0.71	0.68
Phenylalanine + Tyrosine	60.00	93.00	72.68	80.07	76.81	1.21	1.33	1.28	0.78	0.86	0.83
Threonine	40.00	47.00	35.52	38.73	36.71	0.89	0.97	0.92	0.76	0.82	0.78
Valine	50.00	66.00	34.78	37.45	35.64	0.70	0.75	0.71	0.53	0.57	0.54
EAAI			76.95	82.72	79.84						

T.Z, Zhoushan hairtail; T.N, Hainan hairtail; T.Q, Qingdao hairtail.

### Mineral substance

Table 5 shows the concentrations of 12 essential mineral elements found in the muscle of hairtail. According to Afonso et al. (22) and Biandolino et al. (23), potassium, sodium, and phosphorus are the macrominerals with the highest concentrations in the three hairtail samples. In terms of the macroelements studied, potassium (3137.22 mg⋅kg^–1^), sodium (1633.34 mg⋅kg^–1^), and phosphorus (801.19 mg⋅kg^–1^) concentrations were significantly higher in T.N than in the other two varieties. T.Z (243.31 mg⋅kg^–1^) and T.N (296.17 mg⋅kg^–1^) had the lowest and highest calcium concentrations, respectively. Potassium is the most abundant intracellular ion in fish and is essential for fish physiology (e.g., muscle function and membrane potential) (24). These hairtail have a diverse diet, feeding on everything from plankton crustaceans to swimming animals, allowing them to accumulate minerals from a variety of sources. Plankton, for example, is expected to be a good source of phosphorus for upper trophic levels, whereas crustaceans and fish, respectively, are good sources of calcium and potassium (22, 25). Iron (37.63–26.36 mg⋅kg^–1^) and zinc (3.74–3.60 mg⋅kg^–1^) had the highest concentrations of the microelements studied. Copper was the least abundant mineral studied, with 0.35 mg⋅g^–1^ (T.Z), 0.32 mg⋅kg^–1^ (T.N), and 0.27 mg⋅kg^–1^ (T.Q), respectively. Mineral content differences in hairtail of different source have been attributed to a combination of specific characteristics and physiological requirements, diet composition, and mineral bioavailability in the surrounding environment (water and sediments) (26).

**TABLE 5:** Mineral content of three hairtail muscles (mg⋅kg^–1^, w/w).

Parameter		T.Z	T.N	T.Q
Macroelement	K	2881.55	3137.22	2660.89
	P	1552.86	1633.34	1269.69
	Na	679.99	801.19	571.80
	Mg	296.68	263.93	208.48
	Ca	253.41	296.17	243.31
Microelement	Fe	26.36	37.63	24.55
	Zn	3.60	3.66	3.74
	Al	1.96	1.01	2.11
	As	1.93	2.64	1.57
	Sr	1.48	0.54	0.79
	Cr	0.81	0.85	0.72
	Cu	0.35	0.32	0.27

T.Z, Zhoushan hairtail; T.N, Hainan hairtail; T.Q, Qingdao hairtail.

### Fatty acid composition

**FIGURE 3:**
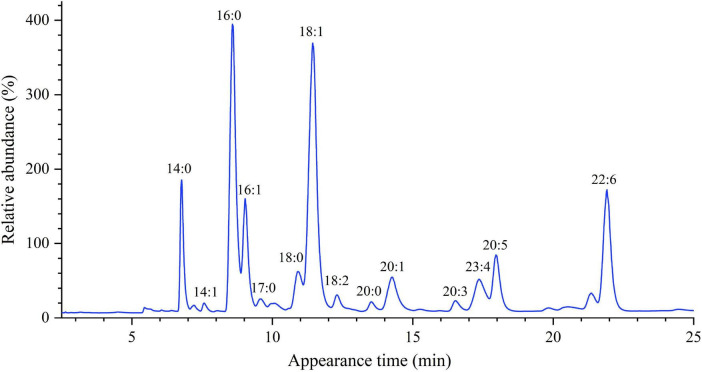
The representative GC chromatogram of hairtail muscle fatty acids.

The fatty acyl composition of the three hairtail samples was investigated to determine the effect of origin on fatty acid selectivity. The FAMEs were analyzed by GC after esterification; the characteristic chromatogram of each fatty acid molecular species is shown in Figure 3. Table 6 summarizes the detection and identification of 16 FAMEs. Overall, the most abundant fatty acid was oleic acid (C18:1) (26.38–28.86%). An OA-rich diet has been shown to improve inflammatory-related disorders, have anti-tumor activity, and lower hypercholesterolemia levels (27, 28). The trans fatty acid (TFA), associated with the increased risk of coronary heart disease and altered prostaglandin metabolism, was not observed in these fishes (29).

**TABLE 6:** Hairtail muscle fatty acid composition from three different sources (%).

Fatty acid	T.Z	T.N	T.Q
C14:0	5.83 ± 0.11^a^	3.56 ± 0.05^b^	3.58 ± 0.02^b^
C15:0	0.49 ± 0.07^a^	n.d.	n.d.
C16:0	22.66 ± 0.21^c^	24.19 ± 0.55^b^	25.41 ± 0.51^a^
C17:0	0.51 ± 0.01^c^	1.26 ± 0.15^b^	1.13 ± 0.01^a^
C18:0	3.03 ± 0.06^c^	5.47 ± 0.41^b^	6.12 ± 0.10^a^
C20:0	0.64 ± 0.02^a^	n.d.	n.d.
SFA	33.16 ± 0.21^c^	34.48 ± 0.07^b^	36.23 ± 0.57^a^
C14:1	0.24 ± 0.02^b^	0.58 ± 0.02^a^	0.55 ± 0.01^a^
C16:1	7.65 ± 0.08^b^	5.41 ± 0.33^a^	6.09 ± 0.11^a^
C18:1 *cis*	28.86 ± 0.39^a^	28.11 ± 0.24^a^	26.38 ± 0.51^b^
C20:1	4.22 ± 0.10^a^	n.d.	n.d.
C24:1	n.d.	n.d.	1.43 ± 0.08^a^
MUFA	40.97 ± 0.35^a^	34.10 ± 0.21^b^	34.45 ± 0.43^b^
C18:2 *cis*	1.26 ± 0.09^a^	0.62 ± 0.02^b^	0.63 ± 0.03^b^
C20:3 n-3	0.88 ± 0.01^c^	1.34 ± 0.08^b^	2.55 ± 0.05^a^
C20:4	4.35 ± 0.02^a^	n.d.	n.d.
EPA n-3	5.48 ± 0.14^a^	4.17 ± 0.23^b^	3.80 ± 0.26^b^
DHA n-3	13.90 ± 0.22^c^	25.28 ± 0.45^a^	22.32 ± 0.34^b^
ΣEPA + DHA	19.38 ± 0.36^c^	29.45 ± 0.45^a^	26.12 ± 0.33^b^
PUFA	25.87 ± 0.44^c^	31.42 ± 0.36^a^	29.30 ± 0.42^b^
UFA	66.84 ± 0.21^a^	65.52 ± 0.07^b^	63.75 ± 0.57^c^

^a–c^Mean values in the same row (corresponding to the same parameter) not followed by a common letter differ significantly (P < 0.05). T.Z, Zhoushan hairtail; T.N, Hainan hairtail; T.Q, Qingdao hairtail.

For saturated fatty acid (SFA), it was similar in content and the main composition was palmitic acid (C16:0), myristic acid (C14:0), and stearic acid (C18:0). For monounsaturated fatty acid (MUFA), the major component of C18:1 in T.Z was slightly higher than the two others, which was in line with the situation of the content of MUFA, but the proportion in T.Z (40.97%) was prominently higher in T.N (34.10%) and T.Q (34.45%). For polyunsaturated fatty acids (PUFA), its proportion was varied in these fish, in the order of T.N (31.42%) > T.Q (29.30%) > T.Z (25.87%). Long-chain (≥ C20) omega-3 polyunsaturated fatty acids (LC-PUFA, e.g., EPA n-3 and DHA n-3) have health benefits against coronary heart disease, inflammatory diseases (e.g., rheumatoid arthritis and hypertension), some cancers as well as various mental disorders (e.g., schizophrenia, ADHD and Alzheimer’s disease), and essential for the nutrition in brain and retina development of infant (30). Docosahexaenoic acid (DHA n-3) and eicosapentaenoic acid (EPA n-3) were the major component of LC-PUFA for hairtail as a research by Usydus et al. (31). It can be observed that the relative content of EPA and DHA of T.N (29.45%) and T.Q (26.12%) was significantly higher than T.Z (19.38%) in the total lipid profile. The fish do not synthesize these oils; rather, microalgae and other marine microorganisms (e.g., thraustochytrids and some bacteria) are the primary source of omega-3 LC-PUFA incorporated in higher marine animals and ultimately in humans through the consumption of seafood (32). The difference of LC-PUFA composition of the three hairtail samples may be related to their discrepant shift and expansion feeding ecology from zooplankton feeding to swimming feeding (25).

### Flavor volatile compounds

Forty-four volatile compounds were detected, including alcohols (7), aldehydes (8), ketones (11), hydrocarbons (15), aromatic compounds (4). Among them, the compounds associated with fresh seafood flavors are mostly 6-, 8-, and 9-carbon aldehydes, ketones, and alcohols derived from the unsaturated fatty acid characteristic of seafood by lipoxygenase activities. As shown in Table 7, significant differences were observed in the molecular species and their concentrations between samples. The most abundant compounds in all samples were hydrocarbons, but they are generally minor contributors to food flavors because of their high odor threshold (33). Aldehydes showed a wide number of different compounds, characterized by low odor thresholds, which have been described as the main responsible of typical freshest most important contributors to the aroma of fish, associated with strong plastic, fatty, geranium, raw fish, and marine odor (34). Nonanal had the highest percentage of aldehydes volatiles among the samples: 1.92% (T.Z), 2.78% (T.N), and 2.80% (T.Q), respectively. The nonanal is the main volatile product of oleic acid oxidation, which can produce a special odor and is associated with a rotten odor. (E)-2-Octenal was derived from the oxidative breakdown of polyunsaturated fatty acids (PUFAs) of linoleic (C18:2) and linolenic (C18:3) acids producing fatty savory smell with 10.7 lg⋅kg^–1^ of the odor threshold value, which derived mainly from vegetable sources in fish diets. In these samples, (*E*)-2-octenal was only detected in T.N and its percentage content ranked second (2.00%) among the aldehydes of T.N. Furthermore, the flavor contribution of Hexanal, Octanal, and Heptanal in the three samples could not be ignored (concentrations, T.N > T.Q > T.Z): Hexanal is derived from linoleic acid oxidation, having a characteristic fruity odor and taste (35). Octanal could be related to the oxidation of n-9 MUFA and, generally, is associated with a soapy, oily, fatty, and citrus odor (36); while Heptanal has a very strong, fatty, harsh, pungent odor, which provides an unpleasant, fatty taste.

**TABLE 7:** Volatile compounds from three different sources were discovered in hairtail muscle (%).

Classify	Volatile compounds	T.Z	T.N	T.Q
Alcohol	1,3-Dioxan-5-ol, 4,4,5-trimethyl-	0.32 ± 0.02^a^	n.d.	n.d.
	1-Hexanol	n.d.	n.d.	1.61 ± 0.90^a^
	1-Octen-3-ol	0.86 ± 0.12^b^	2.02 ± 1.10^ab^	2.84 ± 0.62^a^
	1-Penten-3-ol	n.d.	1.19 ± 0.25^a^	0.69 ± 0.29^b^
	2-Hexadecanol	0.25 ± 0.12^a^	n.d.	n.d.
	4-Heptanol, 2,6-dimethyl-	0.63 ± 0.20^a^	n.d.	n.d.
	Phenylethyl Alcohol	5.37 ± 0.65^a^	n.d.	n.d.
	Subtotal	7.44 ± 0.91^a^	3.21 ± 1.22^b^	5.14 ± 1.43^ab^
Aldehydes	2-Hexenal, (*E*)-	0.16 ± 0.04^a^	0.18 ± 0.05^a^	0.13 ± 0.01^a^
	2-Octenal, (*E*)-	n.d.	2.00 ± 0.62^a^	n.d.
	Butanal, 3-methyl-	0.42 ± 0.07^a^	n.d.	n.d.
	Heptanal	0.61 ± 0.32^ab^	0.90 ± 0.66^a^	0.57 ± 0.25^a^
	Hexanal	1.37 ± 0.81^b^	1.80 ± 0.99^a^	1.70 ± 0.70^a^
	Nonanal	1.92 ± 0.49^b^	2.78 ± 1.32^a^	2.80 ± 1.19^a^
	Octanal	0.82 ± 0.40^b^	1.26 ± 0.78^a^	0.79 ± 0.45^b^
	Subtotal	5.30 ± 2.06^b^	8.92 ± 4.16^a^	5.99 ± 2.59^b^
Ketones	2,3-Pentanedione	0.70 ± 0.16^a^	0.47 ± 0.23^ab^	0.26 ± 0.20^b^
	2-Heptanone	0.97 ± 0.46^b^	2.39 ± 0.29^a^	1.55 ± 0.95^ab^
	2-Hexanone	n.d.	0.23 ± 0.07^a^	0.13 ± 0.09^a^
	2-Nonanone	0.61 ± 0.15^b^	2.89 ± 0.49^a^	2.07 ± 0.72^a^
	2-Pentanone	0.50 ± 0.34^b^	1.35 ± 0.44^a^	0.69 ± 0.58^b^
	2-Undecanone	0.37 ± 0.01^c^	1.57 ± 0.26^a^	1.19 ± 0.17^b^
	3,5-Octadien-2-one, (*E,E*)-	0.78 ± 0.01^a^	n.d.	0.62 ± 0.07^b^
	3-Octanone	n.d.	0.93 ± 0.46^a^	0.50 ± 0.21^b^
	3-Pentanone	0.70 ± 0.27^a^	n.d.	n.d.
	5-Hepten-2-one, 6-methyl-	0.42 ± 0.01^a^	0.46 ± 0.14^a^	0.35 ± 0.06^a^
	7-Octen-2-one	0.35 ± 0.03^b^	0.69 ± 0.08^a^	0.48 ± 0.14^b^
	Subtotal	5.38 ± 1.41^a^	10.98 ± 0.28^a^	7.85 ± 2.67^ab^
Hydrocarbons	Dodecane	1.75 ± 0.17^a^	0.41 ± 0.06^b^	0.41 ± 0.02^b^
	Heptadecane	1.25 ± 0.75^a^	n.d.	n.d.
	Hexadecane, 2,6,10-trimethyl-	n.d.	1.08 ± 0.60^a^	n.d.
	Nonane	0.22 ± 0.05^b^	1.35 ± 0.76^a^	0.36 ± 0.14^b^
	Octane	n.d.	2.74 ± 1.65^a^	1.16 ± 0.42^b^
	Pentadecane	2.28 ± 0.94^b^	1.29 ± 0.40^b^	5.24 ± 1.74^a^
	Pentadecane, 2,6,10,14-tetramethyl-	n.d.	n.d.	2.94 ± 0.98^a^
	Tetradecane	n.d.	n.d.	0.71 ± 0.06^a^
	Tridecane	0.90 ± 0.26^a^	0.63 ± 0.13^b^	0.59 ± 0.12^b^
	Undecane	0.70 ± 0.23^a^	n.d.	n.d.
	1-Undecene	n.d.	n.d.	1.10 ± 0.64^a^
	Benzene, 1-ethyl-3-methyl-	n.d.	0.42 ± 0.07^a^	n.d.
	Ethylbenzene	1.38 ± 0.22^a^	n.d.	1.18 ± 0.25^a^
	*p*-Xylene	3.55 ± 0.62^a^	2.36 ± 0.43^b^	2.60 ± 0.64^b^
	Styrene	1.94 ± 0.51^a^	1.18 ± 0.17^b^	1.97 ± 0.76^a^
	Subtotal	13.97 ± 3.16^ab^	11.46 ± 1.25^b^	18.26 ± 3.90^a^
Aromatic compounds	10-Heptadecen-8-ynoic acid, methyl ester, (*E*)-	0.23 ± 0.02^b^	0.42 ± 0.12^a^	0.28 ± 0.13^b^
	Furan, 2-ethyl-	n.d.	0.35 ± 0.07^a^	n.d.
	Hexadecanoic acid, ethyl ester	0.20 ± 0.03^a^	0.24 ± 0.14^a^	0.23 ± 0.04^a^
	Pyrazine, 2,5-dimethyl-	0.90 ± 0.09^a^	n.d.	n.d.
	Subtotal	1.32 ± 0.07^a^	1.02 ± 0.23^a^	0.51 ± 0.16^b^

^a–c^Mean values in the same row (corresponding to the same parameter) not followed by a common letter differ significantly (P < 0.05). T.Z, Zhoushan hairtail; T.N, Hainan hairtail; T.Q, Qingdao hairtail.

In fish, the relative mass fraction of ketones was slightly higher than that of aldehydes, but the threshold value was significantly higher than that of isomeric aldehydes. The ketones in T.N (10.98%), T.Q (7.85%), and T.Z (5.38%) primarily contribute to the aroma of fruits and lipids, which were produced by thermal degradation of fatty acids with multiple unsaturated bonds, amino acid degradation, or microbial oxidation. 2-Heptanone has a cheesy ketonic, slightly green waxy, banana fruity aroma, and its relative percentage concentrations in T.N (2.89 percent) and T.Q (2.91 percent) were close but significantly higher (*P* < 0.05) than T.Z (0.61 percent). In terms of a rose- and tea-like flavor, 2-nonanone had a significant (*P* < 0.05) difference in T.N (2.93%) and T.Z (0.97%), and when compared to T.Q (0.69 percent) and T.Z (0.50 percent), 2-Pentanone, a colorless liquid ketone with the odor of fingernail polish or a strong fruity odor, had the highest relative percentage concentrations in T.N (1.35 percent). 3-Octanone has a strong, penetrating fruity odor that is reminiscent of lavender, mushroom, ketonic, cheesy, and moldy with fruity nuance, and its concentrations in T.N (0.93 percent) was significantly higher (*P* < 0.05) than T.Q. (0.50 percent). Because of high odor threshold, alcohols are generally minor contributors to food flavors unless they are unsaturated or present in high concentrations. T.Z had a higher volatile substance relative content of alcohol than the others, owing primarily to the affluent phenylethyl alcohol (5.37 percent) with a pleasant floral odor that was unique to T.Z. These findings, as well as the measured percentage of flavor volatile compounds derived from n-3 or n-6 fatty acids, were consistent with the fatty acid composition of the samples: T.N and T.Z were higher in n-3 fatty acids and PUFAs than T.Q.

### Partial least squares-discriminant analysis

**FIGURE 4:**
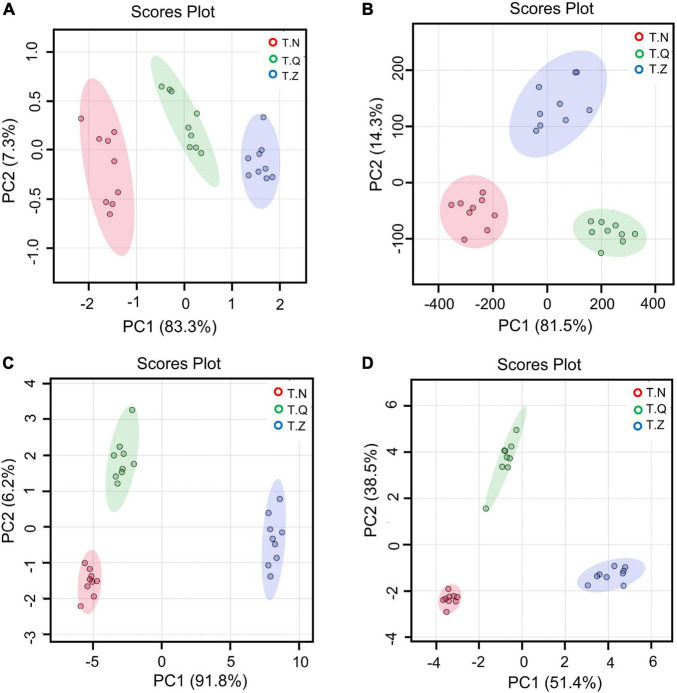
Score plots of PLS-DA of three origin hairtail: **(A)** mineral, **(B)** amino acid, **(C)** fatty acids, and **(D)** volatiles.

Because the color and TPA index could not well distinguish different sources of hairtail in this study, we selected amino acids, minerals, fatty acids and volatile substances as parameters for multivariate statistical analysis to try to find the indicators with the best classification ability. PLS-DA, a supervised discriminant analysis statistical method, uses partial least squares regression to establish a relationship model between the expression of features and sample categories to realize the model prediction of samples (37), showing the differences among three groups of samples. The results are shown in Figure 4. From Figure 4A, the first principal component (PC1) explained 83.3 cumulative percent (cum%) of the variance in the amino acid dataset. Similarly, PC2 explained 7.3 cum%, and PC3 explained 2.0 cum%. Figure 4B showed the PLS-DA of minerals, with PC1 explaining 81.5 cum%, PC2 explaining 14.3 cum%, and PC3 explaining 3.2cum%. From Figure 4C, the PC1 explained 91.8 cum% of the variance in the fatty acids dataset, PC2 explained 6.2 cum%, and PC3 explained 1.0 cum%. In the PLS-DA plot (Figure 4D) regarding flavor substances, the first three principal components could explain 92.7 cum% of the total variance (individual contributions include PC1 explained 51.4 cum%, PC2 explained 38.5 cum%, and PC3 explained 3.0 cum%). When the samples were distributed on different sides of the axis, it indicated that they had been successfully distinguished by the corresponding principal components. The first three principal components of all four sets of models could be considered adequate to demonstrate the variety of different hairtail samples, which shows that all four models can be distinguished well. The performance of the PLS-DA model depends on *R*^2^ (evaluating the fitness) and *Q*^2^ (indicating the predictive ability of the model). Generally, the value of *Q*^2^ more than 0.5 is assigned well for the biological models (38). Among the four models for amino acids, minerals, fatty acids, and volatiles, the value of *Q*^2^ (>0.5) were 0.9598, 0.98220, 97.45, 98.41, respectively; the value of *R*^2^ were 0.9860, 0.9877, 0.9838, 0.9909, respectively. It can be seen that the separation effect of the four groups of model volatile groups was the most obvious, followed by the mineral group.

**FIGURE 5:**
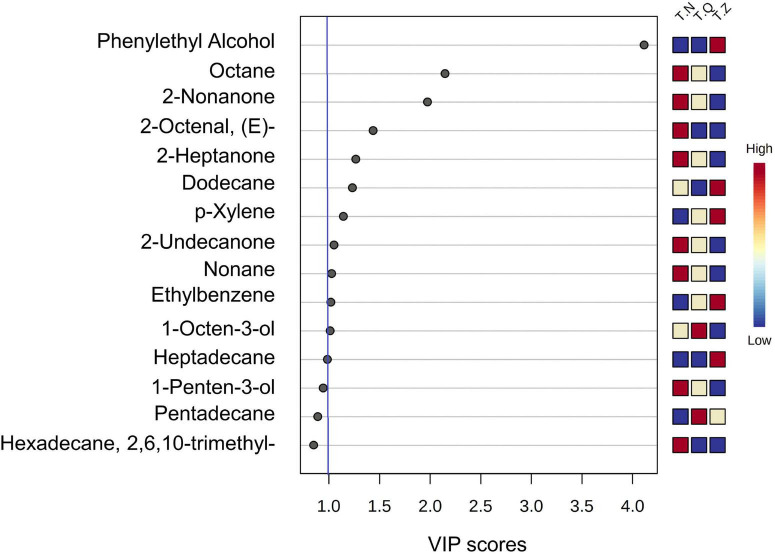
The VIP graph of PLS-DA of all relevant volatiles analyzed in the multivariate data set.

The most prominent variables can be obtained from the VIP plot, which was arranged in decreasing order of VIP, or importance or decreasing order of importance. The variables with VIP > 1 were regarded as effective indicators of class separation (39). In the VIP plot for flavor substances (Figure 5), 11 volatiles, including two alcohols, one aldehyde, three ketones, and five hydrocarbons, were identified as effective in the discrimination of different source hairtail.

## Conclusion

The basic characteristics of hairtail, such as color, texture, nutrient composition, amino acid composition, and volatile flavor substances, differed significantly in this study. T.Z fish caught in the East China Sea had the highest yellowness and crude fat content, with palmitic acid (C16:0) and monounsaturated fatty acids represented by oleic acid being the most abundant fatty acids in lipids (C18:1). T.N fish caught in the South China Sea had the highest concentration of macrominerals (e.g., potassium, sodium, phosphorus, and calcium) and amino acids, the most appropriate proportions of essential amino acids for human nutrition, and their lipids had a high content of PUFA, with the highest contribution of EPA and DHA. Furthermore, the analysis of flavor volatile compounds revealed some differences in the bouquet design of the three samples, particularly in the aroma compounds derived from n-3 or n-6 fatty acids, with more pronounced oil and cheese notes in T.Z and clear and fishy notes in T.N and T.Q, which may influence consumer choice. Other analysis indices, such as fish texture, umami level, and protein content, were largely consistent across the three origin hairtail. In conclusion, the hairtails of three origins can be distinguished based on nutrition, fatty acids, and volatile aroma compounds, which is critical for combating counterfeiting inferiority products and establishing the hairtail brand status in different regions. The classification ability of different indexes for different sources of hairtail were compared and analyzed by multivariate statistical analysis, and it was found that the method of detecting volatile flavor substances by GC-MS was the best way to trace the origin of hairtail. In this study, the most effective method was found among many simple and convenient methods, which enriches the traceability and identification technology of hairtail fish and has high importance to food quality and safety.

## Data availability statement

The original contributions presented in the study are included in the article/supplementary material, further inquiries can be directed to the corresponding authors.

## Ethics statement

The studies involving animals were reviewed and approved by the Ethics Committee of Zhejiang Gongshang University. All applicable international, national, and/or institutional guidelines for the care and use of animals were followed.

## Author contributions

SW: methodology and writing. PW: validation. XS: data curation. HZ: formal analysis. JX: software and validation. KC: conceptualization. QZ, YC, and WL: data curation. QS: conceptualization and funding acquisition. All authors contributed to the article and approved the submitted version.
